# The effect of patients’ preference on outcome in the EVerT cryotherapy versus salicylic acid for the treatment of plantar warts (verruca) trial

**DOI:** 10.1186/1757-1146-5-28

**Published:** 2012-11-12

**Authors:** Sarah Cockayne, Kate Hicks, Arthur R Kangombe, Catherine Hewitt, Michael Concannon, Kim Thomas, Farina Hashmi, Caroline McIntosh, Gwen Brierley, David Torgerson, Ian Watt

**Affiliations:** 1 Department of Health Sciences, York Trials Unit, University of York, York, UK; 2 Division of Podiatry, University of Huddersfield, The School of Human & Health Sciences, Huddersfield, UK; 3 Centre of Evidence Based Dermatology, University of Nottingham, Nottingham, UK; 4 University of Brighton, School of Health Professions, Brighton, UK; 5 The National University of Ireland, Galway, Discipline of Podiatry, Galway, Ireland; 6 Department of Health Sciences, Hull York Medical School, University of York, York, UK

**Keywords:** Randomised controlled trial, Patients’ preference, Plantar warts, Verrucae

## Abstract

**Background:**

Randomised controlled trials are widely accepted as the gold standard method to evaluate medical interventions, but they are still open to bias. One such bias is the effect of patient’s preference on outcome measures. The aims of this study were to examine whether patients’ treatment preference affected clearance of plantar warts and explore whether there were any associations between patients’ treatment preference and baseline variables in the EverT trial.

**Methods:**

Two hundred and forty patients were recruited from University podiatry schools, NHS podiatry clinics and primary care. Patients were aged 12 years and over and had at least one plantar wart which was suitable for treatment with salicylic acid and cryotherapy. Patients were asked their treatment preference prior to randomisation. The Kruskal-Wallis test was performed to test the association between preference group and continuous baseline variables. The Fisher’s exact test was performed to test the association between preference group and categorical baseline variables. A logistic regression analysis was undertaken with verruca clearance (yes or no) as the dependent variable and treatment, age, type of verruca, previous treatment, treatment preference as independent variables. Two analyses were undertaken, one using the health professional reported outcome and one using the patient’s self reported outcomes. Data on whether the patient found it necessary to stop the treatment to which they had been allocated and whether they started another treatment were summarised by treatment group.

**Results:**

Pre-randomisation preferences were: 10% for salicylic acid; 42% for cryotherapy and 48% no treatment preference. There was no evidence of an association between treatment preference group and either patient (p=0.95) or healthcare professional (p=0.46) reported verruca clearance rates. There was no evidence of an association between preference group and any of the baseline variables except gender, with more females expressing a preference for salicylic acid (p=0.004). There was no evidence that the number of times salicylic acid was applied was different between the preference groups at one week (p=0.89) or at three weeks (p=0.24). Similarly, for the number of clinic visits for cryotherapy (p=0.71)

**Conclusions:**

This secondary analysis showed no evidence to suggest that patients’ baseline preferences affected verruca clearance rates or adherence with the treatment.

**Trial registration:**

Current Controlled Trials ISRCTN18994246 and National Research Register N0484189151

## Background

The Randomised Controlled Trial (RCT) is widely accepted as being the ‘gold standard’ design for evaluating the effectiveness of a medical intervention [1]. In an RCT patients are randomly allocated to a study group so that, on average, the groups formed are equivalent in all known and unknown characteristics that may affect outcome. This process should eliminate selection bias, however, it is possible for other types of bias to be introduced after the randomisation process [2-4].

One potential form of bias is that of patients’ preference, which may adversely affect both the external and internal validity of the trial. The external validity of a trial may be affected if large numbers of patients, who have a strong preference for a particular treatment, refuse to take part in the study as they are not guaranteed to receive their treatment of choice [5]. In this case, the generalisabilty of the study’s findings may be limited. The internal validity of a study can also be affected depending on whether or not patients who agree to be randomised receive their preferred treatment. Patients who receive their preferred treatment may be more motivated and comply better with the treatment and report better outcomes compared to those who do not receive their preferred treatment. Furthermore, those patients who do not receive their preferred treatment may experience resentful demoralisation and may be more likely to drop out of the study, resulting in differential loss to follow-up [6].

Whilst the randomisation process will ensure that patients with different treatment preferences are allocated equally between the groups, it is unable to deal with the effect the patients’ treatment preference may have on the outcome measures after randomisation. In order to make comparisons between patients with and without a treatment preference, some trialists have used the patient preference design [7,8]. In this design patients who have a strong preference for one treatment are allocated to their preferred treatment and only patients who are indifferent to which treatment they receive are randomised conventionally. Such a design allows for the examination of patient characteristics associated with preference to be explored. There are however, several limitations to this design. First, it is likely that selection bias will be introduced into the preference arms of the study as they have not been formed by randomisation. Second, it is not known for certain that patients’ preference will affect outcomes, so using this design might mean patients are lost from randomisation needlessly. Finally the design may increase the size and cost of the trial.

An alternative trial design is that of a fully randomised preference trial [6]. In this design all patients who give their consent to participate in the trial are randomised, but their treatment preference is recorded prior to randomisation. This then makes it possible to take treatment preference into account during the analysis of the trial.

Several trials have been undertaken using this approach to assess the effect of patients’ preference on outcome [9,10].

A systematic review and individual patient data meta-analysis in musculoskeletal trials demonstrated that treatment preferences among patients can modify treatment outcomes [11]. To date, the effect of patients’ preference for type of plantar wart treatment on the outcome of the intervention has not been explored. We undertook a randomised controlled trial to assess the clinical and cost effectiveness of cryotherapy using liquid nitrogen and salicylic acid to treat plantar warts in which the patients’ treatment preference was recorded prior to randomisation [12]. The results of the effect of patients’ treatment preference on the outcome of the intervention are reported here.

## Methods

This was a multicentre, two arm randomised controlled open trial. The study was approved by Trent Multicentre Research Ethics Committee (MREC reference 04/mre04/59), Galway Research Ethics Committee and local research ethics committees, Medicines and Healthcare products Regulatory Agency, Irish Medicines Board and local Research and Development Trusts. All patients provided written informed consent prior to being enrolled in the study.

Detailed methods [13] and the main trial results have been published elsewhere [12,14]. In brief, 13 sites within the UK and one in the Republic of Ireland recruited 240 patients from University podiatry schools, NHS podiatry clinics and primary care. Patients were eligible for the study if they were aged 12 years and over and had at least one plantar wart, which in the opinion of the healthcare professional was suitable for treatment with both salicylic acid and cryotherapy. Once written informed consent had been obtained from the patient, baseline data were collected prior to the patient being randomised. This included the treating healthcare professional asking patients whether they had a treatment preference and if so, which treatment did they prefer; salicylic acid, cryotherapy or no treatment preference. Patients were then randomly allocated equally to receive either cryotherapy using liquid nitrogen, for a maximum of four sessions, two to three weeks apart, or daily self-treatment with an over the counter 50% salicylic acid treatment for up to eight weeks. Simple randomisation was used with the allocation sequence generated by a computer which was provided by the York Trials Unit using a remote, secure telephone or on-line randomisation programme. Patients were then followed up at one, three, twelve and twenty-four weeks after entering into the study. The primary outcome was complete clearance of all plantar warts at 12 weeks after randomisation as observed on digital photos by blinded healthcare professionals and by a healthcare professional assessment performed at the recruiting centres. Secondary outcomes for the study included patient self-reported clearance of verruca(e) at 12 and 24 weeks and patient self-reported time to clearance of verruca(e).

All analyses were conducted on an intention to treat basis, including all patients in the groups to which they were randomised. All analyses were conducted in SAS version 9.2 (SAS Institute, NC, USA) and SPSS version 17.0.2 (SPSS) using two-tailed significance tests at the 5% significance level.

The baseline data were summarised descriptively by treatment preference group. The Kruskal-Wallis test was performed to test the association between continuous baseline variables and treatment preference group and to test the association between measures of compliance (number of times salicylic acid applied and attendance at clinic visits for cryotherapy) and preference group. The Kruskal-Wallis test was used as the data did not meet the assumptions for parametric tests. The Fisher’s exact test was performed to test the association between categorical baseline variables and treatment preference group and to test the association between drop out and treatment preference group. The Fisher’s exact test was used as some of the comparisons had less than 80% of expected frequencies greater than 5 and/or at least one expected frequency less than 1. We fitted a logistic regression model with verruca clearance (yes/no) as the primary outcome and treatment, age, type of verruca, previous treatment, treatment preference and the interaction term between randomised treatment and treatment preference as covariates. Two analyses were undertaken, one using the health professional reported outcome at 12 weeks and one using patient’s self reported outcomes at 12 weeks.

Data on whether the patient found it necessary to stop the treatment to which they had been allocated and whether they started another treatment were summarised by treatment group.

## Results

In total 240 eligible patients were recruited to the study between November 2006 and January 2010. One hundred and seventeen patients were allocated to the cryotherapy group and 123 to the salicylic acid group. Two hundred and eighteen patients responded to the question about pre-randomisation treatment preference. One hundred and fourteen (47.5%) patients expressed a treatment preference at baseline; of those 114 patients, twenty-eight (12.8%) expressed a preference for salicylic acid and 86 (39.5%) patients expressed a preference for cryotherapy. One hundred and four (47.7%) patients did not have a treatment preference at baseline. Twenty two (9.2%) patients did not respond to this question. The number of patients allocated to each group along with their treatment preference is summarised in Table 1. The groups were balanced at baseline for treatment preference.

**Table 1:** **Number** (**percent**) **of patients in each treatment group with their treatment preference**

**Treatment preference**	**Treatment randomised**		
	**Cryotherapy**	**Salicylic acid**	**Overall**
Salicylic acid	10 (9.7)	18 (15.7)	28 (12.8)
Cryotherapy	43 (41.8)	43 (37.4)	86 (39.5)
No preference	50 (48.5)	54 (47.0)	104 (47.7)
Total	**103**	**115**	218*

*22 randomised participants had missing preference data.

Tables 2 and 3 present the descriptive statistics for the baseline characteristics of patients and their presenting plantar warts between the three treatment preference groups along with the tests of association. The results from the Kruskal-Wallis test demonstrate that there was no evidence of an association between treatment preference and age (p=0.76), number of verrucae at baseline (p=0.5) and duration of the current verruca (p=0.43). The results from the Fisher’s exact test demonstrate that there was no evidence of an association between treatment preference and type of verruca (p=0.73), previous treatment of verruca (p=0.78), reasons for seeking treatment and type of previous treatment. However, there was evidence of an association between treatment preference and gender (p=0.004), with more females expressing a preference for salicylic acid.

**Table 2:** Baseline characteristics of patients with plantar warts according to treatment preference

**Characteristic**	**Treatment preference**			***P value***
	**Salicylic acid(N**=**28)**	**Cryotherapy(N**=86)*	**No preference(N**=**104)**	
**Age** (**years**)				
Median (interquartile range)	24.8 (16.6, 52.4)	24.2 (17.4, 41.9)	22.9 (18.6, 38.4)	0.76*
**Gender**				
Male	3 (10.7)	37 (44.1)	33 (31.7)	0.004
Female	25 (89.3)	47 (56.0)	71 (68.3)	
Reasons for seeking treatment**				
Painful	11 (39.9)	46 (54.8)	67 (64.4)	0.05
Unable to go swimming	8 (28.6)	21 (25.0)	32 (30.8)	0.69
Unable to participate in other sports	7 (25.0)	17 (20.2)	20 (19.2)	0.82
Other	16 (57.1)	39 (46.4)	40 (38.5)	0.18

Values are n (%) unless otherwise stated.

*2 participants had missing characteristics data.

**Patients may have more than one reason for seeking treatment.

**Table 3:** Baseline characteristics of plantar warts according to treatment preference

**Characteristic**	**Treatment preference**			***P value***
	**Salicylic acid**	**Cryotherapy**	**No preference**	
**Type of verruca**				
Mosaic	5/28 (17.9)	16/86 (18.6)	24/104 (23.1)	0.73
Non-mosaic	23/28 (82.1)	70/86 (81.4)	80/104 (76.9)	
**Duration of verruca** (months)				
N	26	80	100	
Mean(sd)	30.7 (27.5)	22.9 (19.6)	26.6 (27.3)	0.43
Median (interquartile range)	24.0 (12.0, 39.0)	18.0 (9.5, 29.0)	16.8 (8.5, 36.0)	
**Number of verrucae at baseline**				
N	28	86	101	
Mean (sd)	4.3 (4.5)	3.8 (6.6)	3.3 (3.3)	0.50
Median (interquartile range)	3.0 (1.0, 5.5)	2.0 (1.0, 4.0)	2.0 (1.0, 4.0)	
**Previous treatment for current verruca**				
Yes	23/28 (82.1)	66/84 (78.6)	79/104 (76.0)	0.78
No	5/28 (17.9)	18/84 (21.4)	25/104 (24.0)	
Type of previous treatment*				
Self-treatment	21/23 (91.3)	53/66 (80.3)	73/80 (91.3)	0.15
Podiatrist/chiropodist	5/23 (21.7)	23/66 (34.9)	20/79 (25.3)	0.34
GP	10/23 (43.5)	30/66 (45.5)	27/80 (33.8)	0.32
Trial investigating verruca treatments	0/23 (0.0)	2/66 (3.0)	0/79 (0.0)	0.41
Other	3/23 (13.0)	6/66 (9.1)	5/79 (6.3)	0.50

Values are n/N (%) unless otherwise stated.

* Patients may have more than one previous verruca treatment.

When we included an interaction term between randomised treatment and preferred treatment we found no evidence to suggest that patients’ preferences at baseline influenced health professional reported primary outcome (p=0.46) or the patient reported outcome (p=0.95). Overall few patients reported stopping their original treatment (n=21, 11.5%). Of those that did, 16 patients were in the salicylic acid group and five in the cryotherapy group. Only three of the 21 patients started a different treatment (2 in the salicylic acid group and 1 in the cryotherapy group).

Primary outcome data were reported for 229 (95.4%) patients at 12 weeks with 206 (90.0%) having a blinded outcome assessment. Primary outcome data were unavailable for the remaining 11 participants (4 in the salicylic acid group and 7 in the cryotherapy group) due to the patients dropping out of the study. Of these 11, two had their preferred treatment, three did not have their preferred treatment, one did not have a preference and five participants did not express their treatment preferences. There was no evidence of an association between missing data for the primary outcome and whether a participant received their preferred treatment or not (p=0.17).

Table 4 reports the treatment adherence for patients allocated to receive salicylic acid and cryotherapy. There was no evidence that the number of times salicylic acid was applied was different between the preference groups at one week (p=0.89) or at three weeks (p=0.24). There was also no evidence that the number of clinic visits for cryotherapy was different between the preference groups (p=0.71).

**Table 4:** **Treatment compliance** (**number of times salicylic acid applied or number of clinic visits for cryotherapy**)

	**Mean(sd)**	**Median(min,max)**	**p-value**
**Randomised to cryotherapy**			
Preferred cryotherapy	3.58 (0.73)	4 (1, 4)	0.71
Preferred salicylic acid	3.60 (0.52)	4 (3, 4)	
No preference	3.68 (0.79)	4 (1, 5)	
**Randomised to salicylic acid**			
*Week 1*			
Preferred salicylic acid	6.44 (1.03)	7 (4, 7)	0.89
Preferred cryotherapy	6.53 (1.06)	7 (2, 7)	
No preference	6.23 (1.63)	7 (0, 7)	
*Week 3*			
Preferred salicylic acid	6.27 (2.52)	7 (0, 12)	0.24
Preferred cryotherapy	5.79 (3.55)	6 (0, 22)	
No preference	5.04 (2.23)	6 (0, 7)	

## Discussion

The results from this secondary analysis found no evidence to suggest that patients’ baseline treatment preference affected outcome and so receiving the preferred treatment did not confer any additional benefits in those who expressed a preference. The findings of this analysis are not totally unexpected since the primary outcome measure in this study ie clearance of verruca(e) was undertaken by independent blinded healthcare professionals. Such reported measures are unlikely to be influenced by the patients’ preference compared to patient self-reported outcomes other than indirectly through differences in levels of adherence. In order to investigate this further, we explored whether there was an association between (a) healthcare professional reported outcomes and patient treatment preference and (b) patient reported outcomes and treatment preference. In both cases there was no evidence of an association between outcome and treatment preference.

In this study only 114 (52%) patients expressed a treatment preference at baseline. Of these 114 patients, more preferred cryotherapy (n=86) than salicylic acid (n=28) with just over a half of these patients receiving their preferred treatment. Among those with no baseline preference, similar numbers were randomised to the two treatment groups. The difference in treatment preference could be due to several reasons. First, many of the patients who took part in the trial were identified from referrals to cryotherapy clinics. These patients had already decided that cryotherapy was a suitable form of treatment for them and expected this treatment. Secondly whilst we did not collect data on what informed patients’ treatment preference Thomas et al. [15] have reported these findings from a survey of patients within primary care. They reported the most common reason (35%) for patients seeking treatment was to get rid of plantar warts quickly. So patients could have believed that cryotherapy was the more effective form of treatment. Finally, results from the same survey reported the second most common reason for seeking treatment (21%) was due to patients preferring a ‘professional person’ to treat their plantar wart. The fact that both treatments were frequently used within normal practice and were not considered to be novel or new treatments may have contributed towards almost half of the patients (48%) not having a treatment preference. We anticipated that the majority of patients in this study would have received some form of previous treatment prior to taking part in the study and that the efficacy and experiences of these treatment regimens, would inform future treatment preferences. The majority of patients (n = 168, 78%) reported having received some form of previous treatment. Of those 168 patients, 147 (87%) had used some form of self-treatment (salicylic acid or a freezing agent) whilst 48 (29%) and 66 (39%) patients had received treatment from a podiatrist or their GP respectively. There was no evidence of an association between treatment preference and age, duration of verruca, type of verruca and previous treatment of verruca. However, there was evidence of an association between treatment preference and gender, with more females expressing a preference for salicylic acid. As we did not collect data on what informed treatment preference we are unable to report why patients expressed this preference and this could be a chance finding. However, it has been reported that male gender is a predictor for wishing to have a passive role in clinical decision making (i.e., to receive information with a view of following the doctor's advice) [16] and this may tentatively be extrapolated to receiving a clinician delivered treatment.

### External validity

During the course of the study we attempted to collect data on patients who were screened for the study but not enrolled. Two hundred and eighty-four potential patients were approached to take part in the study, 44 of whom were excluded. Approximately half of these (23) were excluded as they were unable to be treated with the trial treatments and only six patients were not interested in taking part in the study. It is possible that data from patients with a strong treatment preference were not collected. For example patients who were approached to take part in the trial whilst requesting treatment at their local GP cryotherapy clinic, may not have been included in the screening data, since sites, understandably concentrated their efforts on collecting data from patients participating in the study. Or patients with strong treatment preferences could have accessed both treatments outside of the study.

The participants in this study had longstanding plantar warts, most of which had been self treated, which is typical of patients presenting to healthcare professionals. These patients were recruited from 14 centres across England, Scotland and Ireland from podiatry clinics, general practice and from the community. We can therefore be confident that the results of this study are broadly generalisable and that the study has external validity across the UK and Ireland.

### Internal validity

There was a low incidence of patients stopping their original treatment and of seeking alternative treatments. Only 21 patients reported stopping their treatment. Since only three (15%) of these 21 patients reported starting another treatment it is unlikely that dilution bias has been introduced. In addition patients who did not receive their preferred treatment did not have higher attrition rates than those who did receive their preferred treatment. We found no evidence to suggest that patients who expressed a treatment and received their preferred treatment adhered to their treatment better than those who did not receive their preferred treatment.

### Comparison with other studies

The proportion of patients who expressed a treatment preference in this study is similar to the median preference rate of 56% reported in a systematic review of patients’ preferences within randomised trials [11]. Unlike our study which found no evidence to suggest that patients’ treatment preference affected outcome, this review concluded that patients’ preferences for treatment may influence outcome [10,11]. However, the review differed from our study in two ways. First the therapeutic area of the review, which focused mainly on musculoskeletal trials, was different to that reported here. Secondly, the review only included trials reporting self-reported outcomes as the primary outcome, whereas the primary outcome for this trial was healthcare professional reported clearance of verrucae. Trials using self-reported outcomes may be expected to be more influenced by patients’ preference as opposed to outcomes reported by blinded independent clinicians, or clearance of disease as used in the EverT trial.

### Strengths and limitations

As far as we are aware, this is the first randomised controlled trial assessing the effect of patients’ preference on the clearance of verrucae. Our study does have some potential limitations. First this study was not powered to detect a difference in preference-treatment interaction and so there may have been insufficient patients in the study to detect a difference, if one existed. Nevertheless, the results of this study could be included with other studies in a meta-analysis to assess the effect of preferences on outcome. Second, the patient’s treatment preference was elicited by the treating healthcare professional, so there was the possibility that their views, (either knowingly or unknowingly) could influence the patients. Care had to be taken when eliciting a response about preference that patients understood and agreed to being randomly assigned to either group. Expressing a particular preference for a treatment would not necessarily result in them receiving the treatment. In order to minimise the possibility of this occurring, clinicians were made aware of this potential limitation during the trial training.

## Conclusions

This trial showed no evidence that treatment preference affected outcomes in the EverT trial. The method employed in this study to explore the effect of patients’ treatment preference on outcome could be used in other trials, and may be a more straightforward alternative to the more complex ‘patient preference’ trial design. Results of this and other trials could then be combined in further work to assess the treatment effects of patients’ preferences in different clinical conditions.

## Abbreviation

RCT: Randomised controlled trial.

## Competing interests

All authors have completed the Unified Competing Interest form at http://www.icmje.org/coi_disclosure.pdf (available on request from the corresponding author) and declare: SC, KH, GB, KT, FH and DT all received proportions of their salaries from the NIHR HTA grant in order to conduct the study. MC, CM and FH’s institutions received funding from the NIHR HTA grant to cover the cost of treating trial participants. All other authors declare no support from any organisation for the submitted work. The 50% salicylic acid (Verrugon) plasters and felt pads were provided to the University of York, free of charge, by the manufacturer William Ransom & Son Plc. BOC Cryospeed provided liquid nitrogen storage equipment at reduced cost. Neither company has had any input into the design, analysis and reporting of the study.

## Authors’ contributions

DT and Jill Hall wrote the original protocol. SC, FH, DT and KT were co-applicants on the application to Health Technology Assessment Programme and refined the protocol. ARK and CH act as guarantors for the paper. DT and IW were Chief Investigators and oversaw the study. SC and KH were the trial coordinators and GB the trial support officer who managed the trial on a day to day basis, assisted, ARK and CH with data validation and data cleaning. CM, FH and MC recruited patients to the study and collected the trial data. ARK and CH designed and conducted the clinical analysis. The initial draft of the manuscript was written by SC and ARK but CH, CM, DT, FH, IW, KH, KT, MC have been involved in revising it critically for important intellectual content. All authors read and approved the final manuscript.
